# The coil orientation dependency of the electric field induced by TMS for M1 and other brain areas

**DOI:** 10.1186/s12984-015-0036-2

**Published:** 2015-05-17

**Authors:** Arno M Janssen, Thom F Oostendorp, Dick F Stegeman

**Affiliations:** Department of Neurology, Radboud University Medical Centre, Donders Institute for Brain, Cognition and Behaviour, Reinier Postlaan 4, 6525 CG Nijmegen, The Netherlands; Department of Cognitive Neuroscience, Radboud University Medical Centre, Donders Institute for Brain, Cognition and Behaviour, Nijmegen, The Netherlands

**Keywords:** TMS, Brain stimulation, Electric field, Coil orientation

## Abstract

**Background:**

The effectiveness of transcranial magnetic stimulation (TMS) depends highly on the coil orientation relative to the subject’s head. This implies that the direction of the induced electric field has a large effect on the efficiency of TMS. To improve future protocols, knowledge about the relationship between the coil orientation and the direction of the induced electric field on the one hand, and the head and brain anatomy on the other hand, seems crucial. Therefore, the induced electric field in the cortex as a function of the coil orientation has been examined in this study.

**Methods:**

The effect of changing the coil orientation on the induced electric field was evaluated for fourteen cortical targets. We used a finite element model to calculate the induced electric fields for thirty-six coil orientations (10 degrees resolution) per target location. The effects on the electric field due to coil rotation, in combination with target site anatomy, have been quantified.

**Results:**

The results confirm that the electric field perpendicular to the anterior sulcal wall of the central sulcus is highly susceptible to coil orientation changes and has to be maximized for an optimal stimulation effect of the motor cortex. In order to obtain maximum stimulation effect in areas other than the motor cortex, the electric field perpendicular to the cortical surface in those areas has to be maximized as well. Small orientation changes (10 degrees) do not alter the induced electric field drastically.

**Conclusions:**

The results suggest that for all cortical targets, maximizing the strength of the electric field perpendicular to the targeted cortical surface area (and inward directed) optimizes the effect of TMS. Orienting the TMS coil based on anatomical information (anatomical magnetic resonance imaging data) about the targeted brain area can improve future results. The *standard* coil orientations, used in cognitive and clinical neuroscience, induce (near) optimal electric fields in the subject-specific head model in most cases.

**Electronic supplementary material:**

The online version of this article (doi:10.1186/s12984-015-0036-2) contains supplementary material, which is available to authorized users.

## Background

Transcranial magnetic stimulation (TMS) [1] is a noninvasive brain stimulation technique that is used in a wide range of neurophysiologic and clinical studies to measure or change the excitability of specific brain areas. Although the popularity of TMS is growing, the mechanism by which the induced electric field affects neuronal excitability is not clear. This holds particularly for the effect of the direction of the induced field relative to the cortical structures. It already has been proven that the effectiveness of the stimulation depends highly on the coil orientation relative to the tissue distribution below the coil [2-4]. Many non-motor brain areas are studied with TMS nowadays [5-10] and general rules about optimal coil orientation, applicable all over the cortex, would help future studies.

A suitable method to obtain knowledge about the induced field and its direction is volume conduction modeling [11-14]. Although several TMS modeling studies have been published in the past 2 decades [12,15-18], the effect of coil orientation on the electric field distribution has not been studied extensively, except for the motor cortex (M1) [19]. Because we are interested in generalizations about coil orientation, the present study concerns the effect of coil orientation also for cortical areas other than M1. For this, the finite element method (FEM) was used. On the basis of agreed optimality for M1 [19,20], the aim was to determine the effect of coil orientation for multiple cortical target sites and the importance of an optimal coil orientation. Generalizations for all cortical areas about the effects of coil orientation were made and the optimality of *‘standard’* TMS coil orientations, used in several cognitive and clinical neuroscience studies, were considered for our subject-specific volume conduction model.

### Optimality and the cortical cosine model

For M1 there is already ample evidence for the importance of coil orientation [2-4,21]. The optimal orientations for this cortical area were determined by finding the highest or most stable motor evoked potential (MEP) amplitude per individual. In general, the optimal coil orientation for M1 induces a primary electric field directed at an angle of approximately 45 degrees to the medial-sagittal plane of the subjects head [2,3]. This orientation induces a posterior-anterior (P-A) directed electric field perpendicular to the central sulcus.

The most logical explanation for the coil orientation preference of M1 stimulation is given by the theoretical *cortical column cosine model of TMS efficacy (C*^*3*^*-model)* [20]. This model is based on the cortical column [22,23] as the functional unit. The authors state that the cortical-column aligned electric field (perpendicular to and directed into the cortical surface) contributes most to the TMS-induced brain activation, due to the fact that the field will be longitudinal and orthodromic to the greatest possible number of cortical neurons at the site of interest. The *C*^*3*^*-model* is supported by volume conduction modeling [19], supported with TMS-positron emission tomography (PET) experiments [20,24], and is nicely in agreement with the orientation specificity found for M1 [2,3].

Due to a lack of an outcome measure like the MEP for cortical target areas outside M1, the optimal orientation cannot easily be obtained experimentally. Nevertheless, several brain structures have been studied with TMS in the course of years [5-10]. The *C*^*3*^*-model* can possibly contribute in determining the optimal coil orientation for these brain areas and improve experimental TMS studies. If the *theoretical* model is applicable to M1, it could be argued that it could as well be applicable to other cortical areas, due to the fact that a similar basic columnar structure can be found all over the cerebral cortex [22,23]. This statement is supported by the orientation specificity found for the supplementary motor area (SMA) [25]. The coil orientation over the SMA that optimally affects the motor output measured with electromyography (EMG) over M1, induces an electric field directed perpendicular to the midsagittal plane and thus perpendicularly into the underlying cortical surface. This TMS coil orientation preference for SMA was verified in a TMS-PET study [26].

Based on the premise that the *C*^*3*^*-model* is applicable to all cortical areas, we determined the effect of coil orientation for thirteen cortical target locations outside M1 in a realistic head model. From the results, generalizations about coil orientation applicable to all cortical target areas are made to predict the optimal orientations.

## Methods

In order to study the induced electric field in the brain, a highly realistic head model with intricate geometrical tissue boundaries was constructed. Herein, fourteen cortical target locations were selected, including M1 (Table 1). The cortical locations were based on clinical and cognitive studies (*references* Table 1). The coordinates for eleven out of these fourteen locations were acquired with the Localite neuronavigational system (http://www.localite.de) from the subject on whom the head model is based. The coordinates for the three other cortical sites were based on visual inspection of the model. For each cortical target location the coil was rotated systematically in steps of 10 degrees (thirty-six orientations in total), while keeping the horizontal plane of the TMS coil at the same level and the center at the same location.

**Table 1:** **Cortical target locations**

**Cortical location**	**Current direction in brain for ‘standard’ orientation**	**Code**
M1 right hemisphere	experimentally determined (highest MEP amplitude)	MR
Lateral cerebellum left [8,40]	rostral (upwards)	CL
Medial cerebellum [8,40]	rostral (upwards)	CM
Lateral cerebellum right [8,40]	rostral (upwards)	CR
O1 (occipital lobe left hemisphere) [9]	medial-lateral	OL
Oz (medial occipital lobe) [9]	medial-lateral leftwards	OM
O2 (occipital lobe right hemisphere) [9]	medial-lateral	OR
Dorsolateral premotor cortex left hemisphere [7,41]	antero-medial	PML
Dorsolateral premotor cortex right hemisphere [7,41]	antero-medial	PMR
Dorsolateral prefrontal cortex left hemisphere [5] (visual)	antero-medial	PFL
Dorsolateral prefrontal cortex right hemisphere [5] (visual)	antero-medial	PFR
Supplementary motor area 30 mm anterior to Cz [6,25]	medial-lateral leftwards	SM1
Supplementary motor area 50 mm anterior to Cz [7,25]	medial-lateral leftwards	SM2
Inferior frontal gyrus [10] (visual)	antero-medial	IL

The cortical target locations commonly used in clinical and cognitive studies, based on neuronavigational data and visual inspection of the model (as indicated). The stimulation locations are based on studies indicated by the references. All cortical target locations are situated in the sulcal wall.

An extensive description of both head model and theoretical background of TMS is provided by Janssen et al. (2013) [14]. The construction of the model will only be described briefly in the paragraph *Volume conduction model*. The induced electric field was computed for all combinations of cortical target site and coil orientation using the FEM (*Theoretical background of TMS*). The FEM was used, because it has been proven to calculate the TMS-induced electric field relatively fast and accurately in a highly realistic anisotropic head model [13,14,27]. At each target location the fields for all coil orientations were compared, as described in paragraph *Data analysis*.

### Volume conduction model

**Figure 1:**
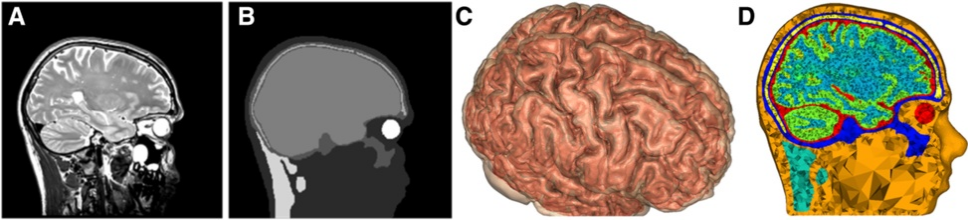
Realistic head model: **(A)** A sagittal cut plane of the T2 weighted MRI showing the different skull layers. **(B)** The same sagittal cut plane of the manually corrected segmentation including skin, skull compacta, skull spongiosa, neck muscle, eyes and one compartment for inner skull (CSF, GM and WM, before segmentation with Freesurfer). **(C)** High resolution triangular surface meshes of GM (transparent) and WM (red), constructed with Freesurfer. **(D)** Sagittal cut plane of the final tetrahedral volume mesh created with TetGen. The different tissue types are represented with different colors.

A prerequisite for studying the effect of coil orientation are realistically described tissue boundaries, and especially the boundary between cerebrospinal fluid (CSF) and grey matter (GM), as introduced in the latest models [12,14]. Spherical volume conduction models [15,16] lack cortical curvature that make it impossible to describe the electric field in the sulci. We therefore incorporated precise geometrical detail and specifically a highly realistically described CSF-GM boundary (Figure 1C). Other important factors are tissue heterogeneity [16] and brain anisotropy [13].

The realistic head model includes eight different tissue types (skin, skull spongiosa, skull compacta, neck muscle, eye, CSF, GM and white matter (WM)) and is based on T1 and T2 magnetic resonance images (MRI) of a healthy 25-year old male subject, with 1 mm^3^ resolution (Figure 1A). The corresponding bulk conductivity values were assigned to different tissue types as previously described [14], σ_skin_ = 0.465, σ_spongiosa_ = 0.025, σ_compacta_ = 0.007, σ_neck muscle_ = 0.400, σ_eye_ = 1.500, σ_csf_ = 1.650, σ_gm_ = 0.276, σ_wm_ = 0.126 (Figure 1D). The head model includes diffusion tensor imaging (DTI) based brain anisotropy, using the volume-normalized approach as described in Opitz et al. (2011) [13]. The model used in this study differs slightly from the one described in Janssen et al. (2013) [14], because in the present study the cerebellum and a detailed GM-WM boundary surface were included as well.

### Theoretical background of TMS

For each combination of cortical target site (Table 1) and coil orientation the induced electric field follows in the quasi-static approach from a subset of the Maxwell equations:

1 $$ \overrightarrow{E} = - \frac{d\overrightarrow{A}}{dt} - \overrightarrow{\nabla}\Phi = - {\overrightarrow{E}}_p - {\overrightarrow{E}}_s $$

with $\overrightarrow{A}$ being the magnetic vector potential, Φ the electrical potential and the $\overrightarrow{E}$ the induced electric field. In the quasi-static approach displacement currents are neglected, which is justified for the stimulation frequency range of TMS (~1–10 kHz). Within this frequency range, the permittivity values for healthy human tissue (within the head) are approximately between 10^3^ε_0_ and 10^5^ε_0_ [28,29], with ε_0_ the permittivity for free space. Previous FEM simulations already demonstrated that permittivity values between 10^2^ε_0_ and 10^4^ε_0_ had negligible effects on the distribution of the induced electric field and only permittivity values in the range of 10^7^ε_0_ had an effect on the electric field distributions [17].

Equation (1) consists of two semi-independent parts. The first part $\frac{d\overrightarrow{A}}{dt}$, which is completely determined by the geometry of the TMS coil and the current strength passing through the coil, is called the primary field (${\overrightarrow{E}}_p$). The second part $\overrightarrow{\nabla}\Phi$, which describes the charge accumulation at conductivity discontinuities in the volume mesh, is called the secondary field (${\overrightarrow{E}}_s$).

The calculation of ${\overrightarrow{E}}_p$ was performed with a custom written C++ program, using an accurate description of a figure-of-eight coil geometry [14,18]. The field distribution of ${\overrightarrow{E}}_p$ was scaled for each combination of target site and coil orientation, such that the maximum field strength just beneath the coil center was 300 V/m.

The secondary field (${\overrightarrow{E}}_s$) depends on the primary field (${\overrightarrow{E}}_p$), the geometry of the volume conductor and its conductivities, and is computed by using the FEM. We used the FEM, because it is able to rapidly compute the induced electric field for a realistic head model with complicated geometrical tissue boundaries (approx. 2.5 minutes with SCIRun^a^ on a Mac Pro, 2.66 GHz Quad-Core Intel Xeon with 16 GB memory). To determine the value of Φ throughout the whole volume mesh, four properties were used: 1.) The induced currents follow Ohm’s law ($\overrightarrow{J} = \sigma \overrightarrow{E}$). 2.) In the quasi-static limit the divergence of the induced current density is zero ($\overrightarrow{\nabla}\cdot \overrightarrow{J} = 0$). 3.) No current leaves the head ($\overrightarrow{J}\cdot \overrightarrow{n} = 0$) (Neumann boundary condition). 4) The induced current density is continuous throughout the volume conductor (${\overrightarrow{J}}_1\cdot {\overrightarrow{n}}_1 = {\overrightarrow{J}}_2\cdot {\overrightarrow{n}}_2$). The resulting system of linear equations was solved with a preconditioned Jacobi conjugate gradient method yielding residuals < 10^−15^. The gradient of Φ was used in combination with the primary field ${\overrightarrow{E}}_p$ to calculate the total electric field for each element inside the head model using equation (1).

### Data analysis

For each combination (target site & orientation), the induced electric field was calculated throughout the whole head model. As we are interested in the TMS induced effects at the cortical level, the fields at the CSF-GM boundary have been visualized. To quantify the effects of coil orientation on the TMS induced field, we used the electric field strength $\left|\overrightarrow{E}\right|$ and the field strength perpendicular to the CSF-GM boundary *E*_⊥_. As stated earlier, the stimulation is probably most effective when the field is perpendicular to the cortical column. This choice was based on the *C*^*3*^*-model,* which can be expected to be applicable to all cortical areas, due to the fact that a similar basic columnar structure can be found all over the cerebral cortex [22,23]. The value for *E*_⊥_ is calculated as ${E}_{\perp } = \overrightarrow{E} \cdot \overrightarrow{n}$, where $\overrightarrow{E}$ is the induced electric field and $\overrightarrow{n}$ the normal vector for the nearest boundary surface triangle.

The target regions, which are used for analysis, are chosen to be spherical (3 mm radius) with their centers located on the cortical surface. By using a fixed radius for each target region, a similar volume is taken for each location. For all targets, except the cerebellar ones, the center of the target region was located in a sulcal wall. They were located in the sulci, because there the field is mostly perpendicular and consequently more likely to be first affected by the stimulation (*Discussion*, section *Cortical cosine model and I-waves*). Within the target region only the GM elements are used to determine $\left|\overrightarrow{E}\right|$ and *E*_⊥_, because there is evidence that the first neuronal activation by TMS takes place at GM level [30]. The optimal orientation is defined as the one inducing the highest mean value for *E*_⊥_.

## Results

### The electric field for standard coil orientations

**Figure 2:**
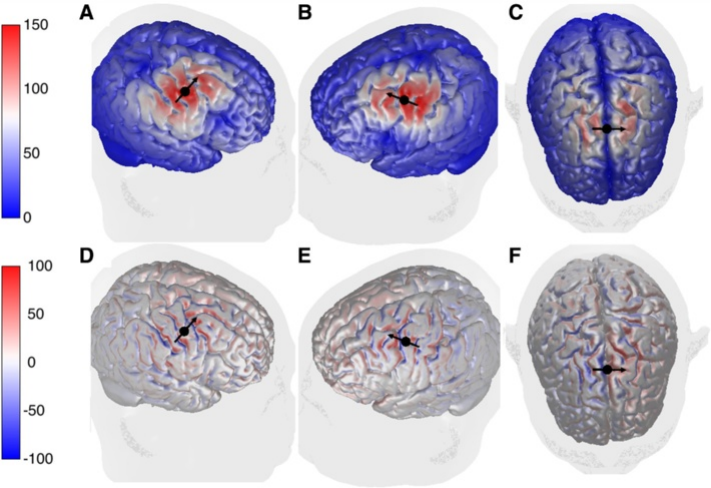
Electric field for three cortical locations: The electric field distribution (V m^−1^), just within the cortex for three locations. On the top row the field strengths $\left|\overrightarrow{\mathrm{E}}\right|$ for **(A)** the right motor cortex (MR), **(B)** the left premotor cortex (PML) and **(C)** the supplementary motor area 3 cm anterior to Cz (SM1) are displayed. In the bottom row the field strengths perpendicular to the CSF-GM boundary E_⊥_ are shown for **(D)** MR, **(E)** PML and **(F)** SM1. For the later scale, a positive value means directed inward and a negative means directed outward. The black dot indicates the location of the center of the TMS coil. The direction of the primary electric field directly under the coil center is indicated with the black arrow.

In Figure 2, the electric fields at the cortical level are visualized for three locations and their corresponding *standard* coil orientations reported in literature (MR: left column, PML: middle column & SM1: right column, Table 1). The electric field strength ($\left|\overrightarrow{E}\right|$, top row) and the field strength perpendicular to the CSF-GM boundary (*E*_⊥_, bottom row) are shown. The black arrow indicates the direction of the primary electric field directly beneath the coil center (*black dot*).

All target locations have multiple gyri with high field amplitudes near the target site (Figure 2, top row, *red*). The highest electric field values are located at the crowns and lips of the gyri, which is in accordance with earlier reports [11,12]. High field values can be found for multiple gyri anterior and posterior to the target site following the midline of the coil (Figure 2, top row, *red and pink*).

The highest field values for the perpendicular component (*cortical column aligned*) were found in the sulci and almost never on top of the gyri (Figure 2, bottom row). A distinction can be made visually between the inward (*red*) and outward (*blue*) directed electric field. The maximum field values for all fourteen target locations and their *standard* coil orientations, determined over the complete cortical surface, are listed in Table 2. The maximum values for *E*_⊥_ are always lower than the maximum values of $\left|\overrightarrow{E}\right|$, as expected. However, the maximum values for *E*_⊥_ are still between 45 and 80 percent of their corresponding maximum value for $\left|\overrightarrow{E}\right|$.

**Table 2:** **Maximum TMS induced electric field**

	**Maximum electric field strength [V m** ^**−1**^ **]**	
	***Ē***	***Ē***_┴_
MR	157.7	86.1
PMR	163.5	101.3
PFR	150.4	81.9
PFL	142.3	100.9
PML	170.7	96.7
IL	142.7	96.6
OL	130.5	82.2
SM2	117.9	93.4
SM1	130.2	104.9
OR	114.8	73.6
OM	124.3	74.2
CL	101.0	49.1
CR	101.9	47.2
CM	95.5	62.3

Values on cortical surface. The maximum values for the electric field strength $\left|\overrightarrow{\mathrm{E}}\right|$ and the field strength perpendicular to the CSF-GM boundary E_⊥_ for all fourteen target locations with the standard coil orientations found in literature. The cortical target location coding can be found in Table 1. The values are based on the complete cortical surface.

### Change in coil orientation for M1 stimulation

**Figure 3:**
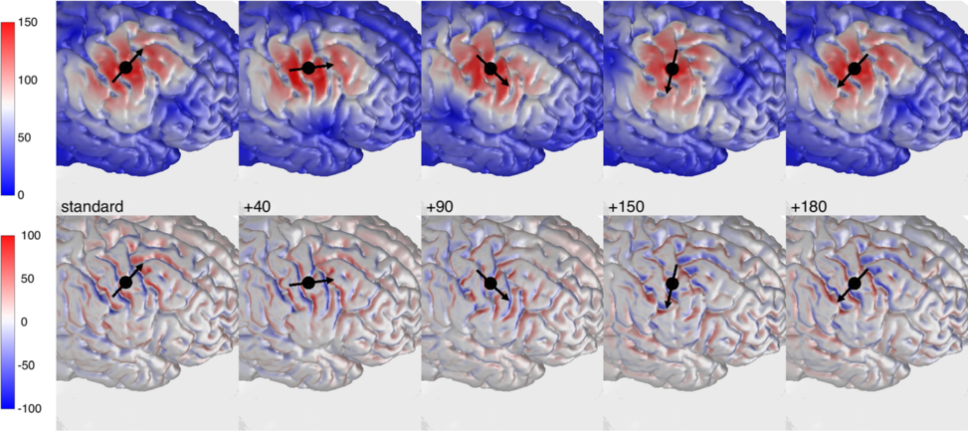
Electric field for five coil orientations over M1: The electric field distribution (V m^−1^), just within the cortex, for M1 stimulation with the standard coil orientation (1st column) and 4 other orientations (+40 (2nd column), +90 (3rd column), +150 (4th column) and +180 (5th column) degrees of clockwise rotation). The field strength $\left|\overrightarrow{\mathrm{E}}\right|$ (top row) and the field strength perpendicular to the CSF-GM boundary E_⊥_ (bottom row) are shown. For the later scale, a positive value means directed inward and a negative means directed outward. The black dot indicates the location of the center of the TMS coil. The direction of the primary electric field directly under the coil center is indicated with the black arrow.

In Figure 3 the results are presented for 5 coil orientations over M1, namely (1) the *standard* from literature, (2) the *standard* + 40 degrees, (3) + 90 degrees, (4) + 150 degrees and (5) + 180 degrees of clockwise rotation. The induced electric field strength ($\left|\overrightarrow{E}\right|$, top row) and the field strength perpendicular to the CSF-GM boundary (*E*_⊥_, bottom row) are shown. The black arrow again indicates the direction of the primary electric field directly under the coil center (*black dot*). Both rows in Figure 3 show the effect of coil orientation on the electric field distribution. The highest electric field values are always located at the crowns and lips of gyri for all orientations (Figure 3, top row). However, no clear orientation dependency can be observed in the field strength on top of the precentral gyrus (M1, *around black dot*).

The component perpendicular to the cortical surface shows no high field values on top of the gyri, but always inside the sulci (Figure 3, bottom row). The field clearly differs between orientations. The consistency of the calculations is expressed by the fact that the absolute strength of the electric field becomes the same for the *standard* orientation and the 180 degrees rotation of the coil; only the direction of the field reverses from inward to outward (*red turns blue and vice versa*). The *standard* orientation induces the strongest *E*_⊥_ values directed into the cortex at the anterior sulcal wall of the central sulcus. This is in accordance with earlier findings [19,20,24].

**Figure 4:**
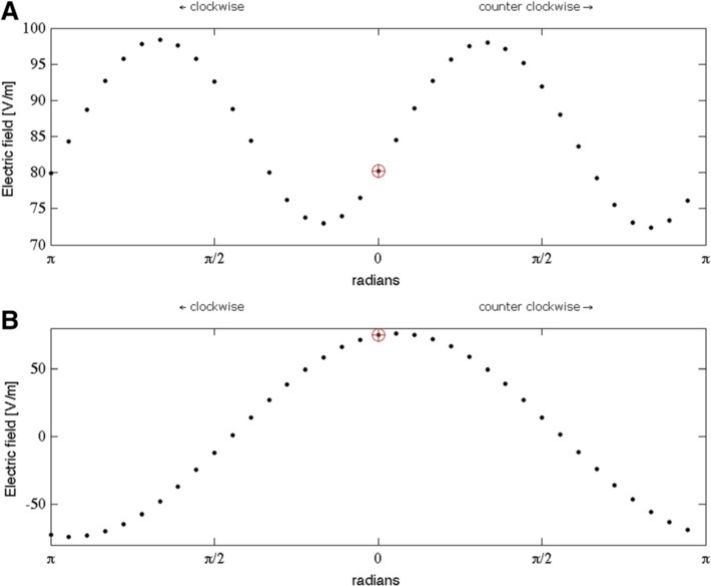
Mean electric field strength in target region M1: The mean electric field values for **(A)** $\left|\overrightarrow{\mathrm{E}}\right|$ and **(B)** E_⊥_ within the target region M1. The standard coil orientation from literature is indicated separately in both panels (red circle with cross). The coil is rotated in steps of 10 degrees.

The results from Figure 4 show that coil orientation has an effect on the TMS induced electric field distribution and therefore probably also on the TMS induced activation of neuronal structures. In Figure 4 the mean values for $\left|\overrightarrow{E}\right|$ and *E*_⊥_ within the target region MR are shown for all thirty-six orientations. The *standard* coil orientation from literature is indicated in both panels of Figure 4 (*red circle with cross*). The results again show that for M1, the orientation dependency of the mean field strength is small (Figure 4A), especially compared to the dependency of the perpendicular electric field (Figure 4B). Based on the mean field strength the *standard* coil orientation induces an electric field far from optimal (Figure 4A, *red circle with cross*). However, the *standard* coil orientation induces almost the highest possible perpendicular electric field, directed into the cortex (Figure 4B *red circle with cross*).

It is known from earlier reports that a coil rotation of 90 degrees (compared to the most optimal orientation) will induce the least effective electric field [3]. The results from this study show that the field perpendicular to the cortical surface is almost equal to zero with both a clockwise or an anti clockwise rotation of 90 degrees. The results presented in Figure 4 are clearly in favor of the argument that the optimal field is directed perpendicular and into the cortical surface as found in previous studies [19,20,24].

### Optimization of stimulation at other locations

Following the same procedure as for M1, the mean values for $\left|\overrightarrow{E}\right|$ and *E*_⊥_ within the target region for all thirty-six orientations over the other thirteen cortical surface targets have been calculated. The mean values for the *standard* coil orientation and the optimal orientation are listed in Table 3 (*per target location*). The results for all other coil orientations can be found in the Additional file 1: Mean electric field strength for all target regions. The optimal orientation for the outcome measures $\left|\overrightarrow{E}\right|$ and *E*_⊥_ are determined separately (Table 3). Also for the other locations the optimal orientation can differ between outcome measures (Additional file 1: Mean electric field strength for all target regions). This means that it is important to choose the optimal orientation, based on the correct outcome measure. Here we decided to use the *C*^*3*^*-model* (*E*_⊥_) [20] as well, because this theory best explains orientation dependency.

**Table 3:** **Mean electric field values for standard and optimal coil orientation**

**Location**	**Mean electric field strength [V m** ^**−1**^ **]**			
	***Ē***_***standard***_	***Ē***_***optimal***_ ***(% standard)***	***Ē***_⊥__***standard***_	***Ē***_⊥__***optimal***_ ***(% standard)***
**MR**	80.3	98.3 (122)	74.9	76.1 (102)
**PMR**	131.5	138.0 (105)	119.0	138.7 (117)
**PFR**	111.7	112.4 (101)	83.8	84.9 (101)
**PFL**	123.7	127.3 (100)	76.2	76.7 (100)
**PML**	107.7	114.3 (106)	40.6	73.6 (181)
**IL**	106.5	115.7 (109)	52.9	52.9 (100)
**OL**	100.6	102.9 (102)	65.0	66.6 (102)
**SM2**	86.7	109.7 (127)	64.9	64.9 (100)
**SM1**	74.4	89.8 (121)	54.2	55.7 (103)
**OR**	80.2	87.0 (108)	64.3	66.6 (104)
**OM**	98.9	99.2 (100)	51.4	51.4 (100)
**CL**	68.6	71.2 (104)	4.9	18.7 (382)
**CR**	81.8	82.2 (100)	−7.2	9.3 (−129)
**CM**	88.4	89.8 (101)	42.7	45.0 (105)

The mean electric field values for $\left|\overrightarrow{\mathrm{E}}\right|$ and E_⊥_ within the target region. For each location the value for the standard coil orientation and the optimized coil orientation are given. The optimized values are determined for both outcome measures ($\left|\overrightarrow{\mathrm{E}}\right|$ and E_⊥_) individually. The cortical target location coding can be found in Table 1.

**Figure 5:**
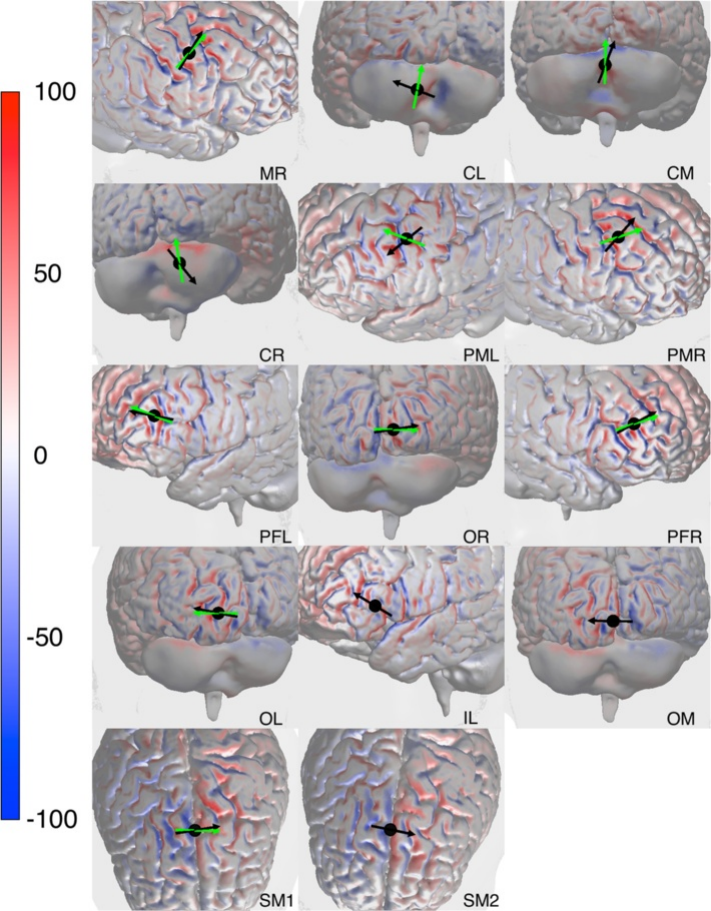
Optimal coil orientation for all target locations: The optimized electric field perpendicular to the CSF-GM boundary E_⊥_ (V m^−1^), just within the cortex, for all fourteen cortical target locations (Table 1). The cortical location index from Table 1 is shown in every right bottom corner. A positive value means directed inward and a negative means directed outward. The black dot indicates the location of the center of the TMS coil. The direction of the primary electric field directly under the coil center is indicated with the black arrow for the optimized coil orientation. The green arrow indicates direction of the primary electric field for the standard coil orientation.

Most of the *standard* orientations found in literature can be considered almost optimal for inducing the strongest perpendicular fields in nearby sulcal walls in our subject-specific model. Only four out of fourteen target locations could possibly be improved with more than 5 percent (PMR, PML, CL and CR). A generalization of the results will be discussed in the paragraphs *Simulation outside M1 & Generalization*. Because of their distinctive results, the cerebellar targets will be discussed separately in more detail in the paragraph *Cerebellum*. The electric field distribution per target location for the optimal coil orientation, which induces the strongest perpendicular field directed into the cortex, is shown in Figure 5.

## Discussion

### Motor cortex

The variation in the induced electric field for M1, caused by a change of coil orientation, has been visualized and quantified. Although the strongest electric field can be found on top of the precentral gyrus for all coil orientations, no clear orientation dependency can be observed in the field strength at this cortical location (Figure 3, top row, *around black dot*). The electric field on top of the gyrus is primarily parallel to the cortical surface and never perpendicular. According to the *C*^*3*^*-model*, the electric field has to be perpendicular and directed into the cortical surface (orthodromic to the underlying cortical neurons [31]).

In the central sulcus, the strength of the perpendicular component varies strongly with coil rotation (Figure 3, bottom row and Figure 4B). The coil orientation dependency of the mean field strength is small in the sulcal wall (Figure 4A). For M1, the strongest perpendicular fields (*positive and negative*) are produced by a coil orientation of 45 degrees relative to the medial-sagittal plane. A 90-degree coil rotation compared to the optimal orientation, which aligns the figure-of-eight midline with the central sulcus, produces a weak perpendicular component (Figure 4B). The results from this study are nicely in agreement with experimental findings [2,3] and previous modeling results of [19]. They confirm that the field in the sulcal wall (and orthodromic to the cortical neurons [31]), is highly susceptible to coil orientation changes and most probably a primary location for neuronal activation.

### Stimulation outside M1

The local anatomy of the areas outside M1 are different compared to M1 and therefore the optimal orientation of the TMS coil has to be determined per target location (Table 1). In general, all locations display multiple gyri with high electric field strengths near the targeted cortical location for all coil orientations. The highest field values are located on top of the gyri, which is similar to the results of M1 and earlier reports [11-14]. Similar to M1, the electric field on top of the gyri is mainly parallel to the cortical surface and therefore probably not susceptible to coil orientation changes. Considerable field values are also found in the sulcal walls, where it is considered to be highly effective due to its direction (*perpendicular to the cortical surface*) (Figure 2 and Figure 5).

To determine whether the *standard* TMS coil orientations (*references* Table 1) can be improved for the subject model at hand, we calculated the field perpendicular to the cortical surface in target regions located in the nearest sulcal walls (*Methods*, *Data analysis*). For almost all cortical target regions chosen in this study the *standard* TMS coil orientations are inducing an (near) optimal electric field (Figure 5 and Table 3). This was not the case for the locations PMR, PML, CL and CR. For PMR and PML a simple coil rotation (−30 and +40 degrees) could be applied to direct the field perpendicular to the sulcal wall in the target region and make it optimal. The results for CR and CL deserve some more attention and are discussed in more detail in paragraph *Cerebellum*.

For the cerebellar (CL, CR & CM) and the DLPMC locations (PMR & PML) the choice of orientation was based on physiological outcome measures. For the SMA locations (SM1 & SM2) the choice of orientation was validated by physiological outcome measures. For the other locations the *standard* TMS coil orientations could be based on the theory that the induced field should be perpendicular to the underlying cortical gyrus. Therefore, one could say that it is not surprising that these coil orientations produce the electric fields with almost the strongest perpendicular component. However, most experimental studies still determine their coil orientation on general landmarks, for example an angle relative to the saggital midline. The standard orientations used in this study are also not based on anatomical MRI data, but on these general landmarks. It is therefore reassuring that the orientations, based on these general landmarks, also produce electric fields with a strong perpendicular field in our subject-specific head model.

### Generalization

Of course, due to the inter-individual differences in head and brain anatomy, the optimal coil orientations found in our model can be sub-optimal for other individuals. Nevertheless, there are still several important conclusions that can be drawn from the results presented. First and most important, the general rule that the figure-of-eight TMS coil has to be oriented perpendicular to the underlying sulcal wall and has to induce an inward directed electric field is also valid for areas outside M1. This means that orienting the coil based on anatomical information about the targeted brain area (for example with anatomical MRI data) can improve the results of the study. Elaborate computational modeling might not be needed to determine the optimal orientation, although it can provide much information about the induced electric field. Secondly, it can be considered reassuring that the *standard* TMS coil orientations appear near optimal for the head model used in this study. This could imply that the inter-individual differences in curvature are small enough to not drastically changing the induced electric field (perpendicular to the cortical surface). However, the specific results for the locations PMR and PML lessen this statement. Third and lastly, the results show that a coil rotation of 10 degrees (*from the optimal orientation*) does not change the electric field much (Figure 4, Additional file 1: Mean electric field strength for all target regions). This means that small orientation errors (for example due to improper placement of the coil by the experimenter) will probably not affect the TMS induced effects much. An orientation error of 90 degrees will definitely minimize the TMS effect, but this kind of error is highly improbable with the neuronavigational tools commonly used today.

### I-waves and the perpendicular electric field

The cortical response to TMS depends on a complex interaction between the applied electric field distribution and the neural elements and networks in the cortex. Herein, the orientation of the electric field is essential, as shown in this study, but also aspects like the type of coil, stimulation (single, paired-pulse or repetitive) and pulse waveform are important.

**Figure 6:**
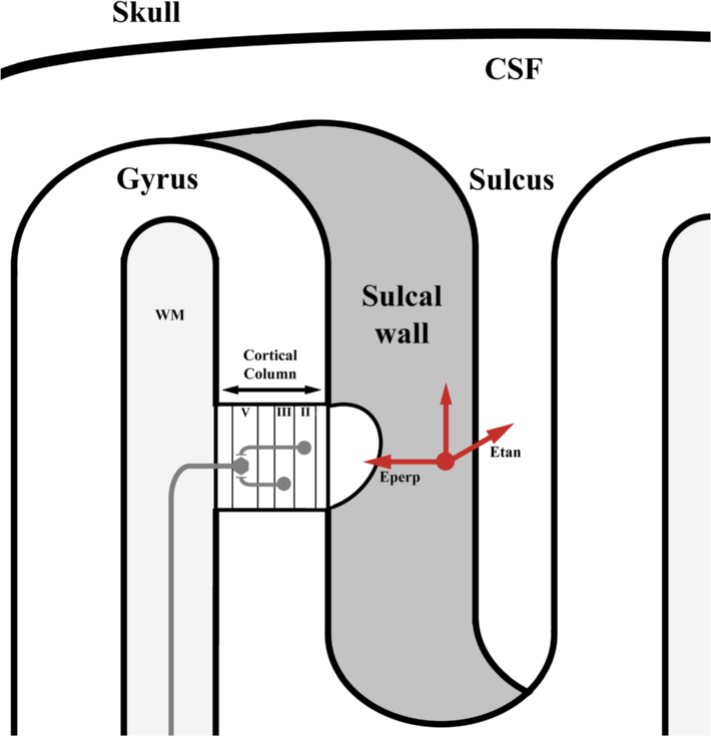
Cortical column in sulcal wall: A simplified schematic representation of the cortical column in the sulcal wall. Included are neural elements (P2, P3, P5) that are possibly stimulated by the electric field component aligned with the axis of the cortical column. The electric fields perpendicular (Eperp) and tangential (Etan) to the sulcal wall are represented by red arrows.

A generally accepted theory to explain the mechanisms of cortical activation in M1 is based on the generation of the direct (D) and the indirect (I) waves. Stimulation of M1 with a figure-of-eight TMS coil, a monophasic waveform and a posterior-anterior (P-A) field direction, produces several I-waves, reflecting the indirect activation of the layer V pyramidal neurons (P5) [30]. With higher intensities direct activation of the P5 neurons is accomplished as well, generating a D-wave. The corticospinal wave with the lowest TMS threshold for this specific type of stimulation is called the I1-wave. The generation of this wave has an orientation preference of the electric field (electric field directed PA to the hand-knob) [32]. The indirect stimulation of layer V pyramidal neurons (P5) in this TMS set-up is probably due to the activation of excitatory pyramidal neurons in layers II (P2) and III (P3) in the cortex [33] (Figure 6).

The P2 and P3 axonal connections to the P5 neurons lie within a cortical column, along the direction of the cortical column axis. This means that an electric field perpendicular to the cortical surface is likely to produce an I1-wave. Because the direction of the induced electric field is predominantly parallel to the plane of the TMS coil, the field in the sulci is mostly perpendicular to the cortical surface. At the top of the gyri the TMS induced electric field is mostly parallel to the cortical surface. This would mean that the I1-wave following TMS stimulation originates in the sulcal wall. The later I-waves are produced by complex circuits, higher stimulation intensities and possibly by other electric field components [32]. This could mean that the electric field direction preference is most applicable to the I1-wave and that the effects of coil orientation are most prominent at low stimulation intensities.

The results for stimulation of M1 with a figure-of-eight TMS coil, a monophasic waveform and a P-A field direction are nicely in agreement with the argument stated above. However, there are also other protocols and TMS hardware set-ups. For example, stimulation with a figure-of-eight coil and a biphasic waveform produces less homogeneous descending cortical volleys compared to stimulation with a monophasic waveform [30,33]. This could mean that also other neural elements are activated by such stimulation. Still, the anterior-posterior-posterior-anterior (AP-PA) orientation produces a similar pattern of recruitment of D and I waves with increasing stimulation intensities as the monophasic PA stimulation [30,33].

The above argument is based on the assumption that cortical activation occurs through stimulation of neural elements aligned with the axis of the cortical column. However, this is certainly not the only possible mechanism of cortical activation. For a detailed discussion about the possible mechanisms of cortical activation and neural elements that can be stimulated by TMS, see for example [34].

### Cerebellum

The results in Table 3 and Figure 5 suggest that the *standard* coil orientation for CR and CL stimulation, which induce an electric field with a caudal-rostral direction, cannot be considered optimal. The optimal orientations found in this study would induce a medial-lateral directed field. In addition, the results from Table 3 suggest that lateral cerebellar stimulation is highly unlikely due to the low values for the perpendicular field. However, it is known from previous studies that the cerebellum can be stimulated [8,35].

There are two possible explanations for the discrepancies. The first reason could be that the neuronal structures in the cerebellum are quite different with their Purkinje cell population. These cells might be stimulated in a different way and more susceptible to an electric field that is directed parallel to the cerebellar surface. A different reason could be the absence of cerebellar gyri and sulci in this particular model. This is due to the fact that the model is based on 3-Tesla MRI in which the cerebellar gyri are too small to be discerned reliably on the MR images. Therefore, we cannot determine a perpendicular component of the electric field in the sulcal walls of the cerebellum. For future modeling studies that particularly focus on the cerebellum, it would be important to include cerebellar gyri in the model construction process.

### Limitations and Validation

The *C*^*3*^*-model* is highly suitable to explain the effect of coil orientation on the activation of neuronal populations, but it is still a simplification of the mechanism responsible for the neural activation by TMS. The parallel component of the electric field might also contribute to the activation of neurons in the cortex. As mentioned earlier in the section *I-waves and the perpendicular electric field*, at higher intensities late I-waves are produced by more complex circuits and possibly other electric field directions [32]. The notion that other electric field directions possibly also contribute to the generation of MEPs is strengthened by the study of Opitz et al. (2013) [36]. Within a specified area of M1, correlations were found between the MEP amplitude and both the mean strength of the perpendicular component as well as the mean tangential component of the electric field. Although these findings appear to be in contrast to the assumption that the perpendicular component is the most important for coil orientation dependency, this is not necessarily the case. The correlations were determined for the variation in MEP amplitude due to coil position and not specifically for coil orientation. The strengths of both electric fields components are likely to depend on the distance to M1, as does the MEP amplitude. It could therefore still be that both electric field components contribute to the generation of MEPs, but that only the strength of the perpendicular component contributes to the orientation dependency.

The results of this study are also based on assumptions and simplifications about neuronal activation for different cortical areas. The most important ones are the similar mode of neuronal activation and the preferred direction of the electric field for all cortical areas. Nonetheless, the distribution or type of neurons may differ and also the preference of direction for activation by the induced electric field (see *Cerebellum*). However, the assumptions are justified by the fact that a similar basic columnar structure can be found all over the cerebral cortex [22,23]. We think that as long as no knowledge is available about the differences in activation mechanisms between cortical areas due to TMS, it is reasonable to assume that the same intensity and direction relative to the CSF-GM boundary is needed to stimulate neuronal populations in all cerebral areas.

The presented FEM simulations are based on well-established laws of physics (*Methods*, section *Theoretical background of TMS*) and the calculated fields are valid. However, the results still have to be verified with careful validation experiments. In these experiments the dependence of the coil orientations should be tested for non-motor brain areas, for example with concurrent TMS-fMRI [37], TMS-EEG [38], phosphene threshold (*occipital cortex*) or with two coil - paired pulse protocols (*cortical areas connected to M1*). Such experiments have already been performed, for example for the SMA [25,26] of which the physiological measurements are in agreement with the results presented here. Nevertheless, to validate the general rules that the induced electric field should always be directed perpendicular to the underlying gyrus and that small orientation changes do not have a large effect on the outcome measures, new validation experiments should be performed. In these experiments, the exact cortical target location should be verified with for example fMRI and the coil orientation should be varied in small 10-degree steps. This way the exact orientation relative to the cortical target can be determined. With these experiments also the justification of the previous mentioned simplifications about neural activation can be tested.

### Future volume conduction models

Previous reports mainly directed their attention on the strength of the electric field and did hardly address the electric field direction [11,12]. Other studies did include direction, but focused only on one sulcus [39,34]. We here want to make an argument for focusing on direction relative to the underlying cortical structures. In this study we decided to focus on the field perpendicular to the cortical surface, based on the *C*^*3*^*-model* [19,20]. A related approach would be to focus on the field direction guided by the first eigenvector of the DTI at the GM-WM interface [36].

Producing complex and realistic finite element models is time-consuming and requires a significant amount of computational power. It is therefore that often spherical or low-resolution models are used instead. However lack of cortical curvature, as in the first spherical models [15,16], makes it impossible to study the electric field within sulci and thereby underestimate the field perpendicular to the cortical surface. It can be concluded that modeling studies should include a realistic CSF-GM boundary to properly answer questions about the induced electric field at the cortical level.

## Conclusions

The effect of coil orientation for multiple cortical target sites was determined and generalizations for all cortical areas were made. In addition, the optimality of *‘standard’* TMS coil orientations used in some example cognitive and clinical neuroscience studies were considered for our subject-specific volume conduction model. The results for M1 are nicely in agreement with experimental findings [2,3] and confirm previous modeling results [19]. For all cortical targets, the electric field perpendicular to the sulcal walls is considered to be the most effective and most susceptible to coil orientation changes. Small coil orientation changes do not alter the induced electric field drastically. We suggest that the general rule to optimize the effect of TMS should be that the strength of the electric field perpendicular to the targeted cortical surface area (and inward directed) has to be maximized. Therefore, orienting the coil based on anatomical information about the targeted brain area can improve future study results (for example with anatomical MRI data). The *standard* TMS coil orientations, based on previous studies, also seem to be near optimal for some cortical target areas in the subject-specific individual head model. This last finding has to be replicated with more than one subject model and the general rules about coil orientation should be validated with experimental studies.

### Endnotes

^a^The freely available SCIRun 4.5 (Scientific Computing and Imaging Institute, Salt Lake City, UT).

## Additional file

Additional file 1:
**Mean electric field strength for all target regions.**

## Abbreviations

TMS: Transcranial magnetic stimulation
FEM: Finite element method
M1: Motor cortex
C^3^-model: Cortical column cosine model of TMS efficacy
PET: Positron emission tomography
MEP: Motor evoked potential
SMA: Supplementary motor area
EMG: Electromyography
CSF: Cerebrospinal fluid
GM: Grey matter
WM: White matter
MRI: Magnetic resonance imaging
DTI: Diffusion tensor imaging
