# Supplementary material for: The coil orientation dependency of the electric field induced by TMS for M1 and other brain areas
Source: J Neuroeng Rehabil. 2015 May 17;12:47. doi: 10.1186/s12984-015-0036-2 (PMC4435642; doi:10.1186/s12984-015-0036-2)

Supplementary Material A:

*The mean electric field values for (**A**) and **(B)** within the target regions (Methods 2.3) defined in Table 1. The standard coil orientation from literature is indicated in both panels (red circle with cross). The coils are rotated in steps of 10 degrees.*

Lateral cerebellum left (CL)

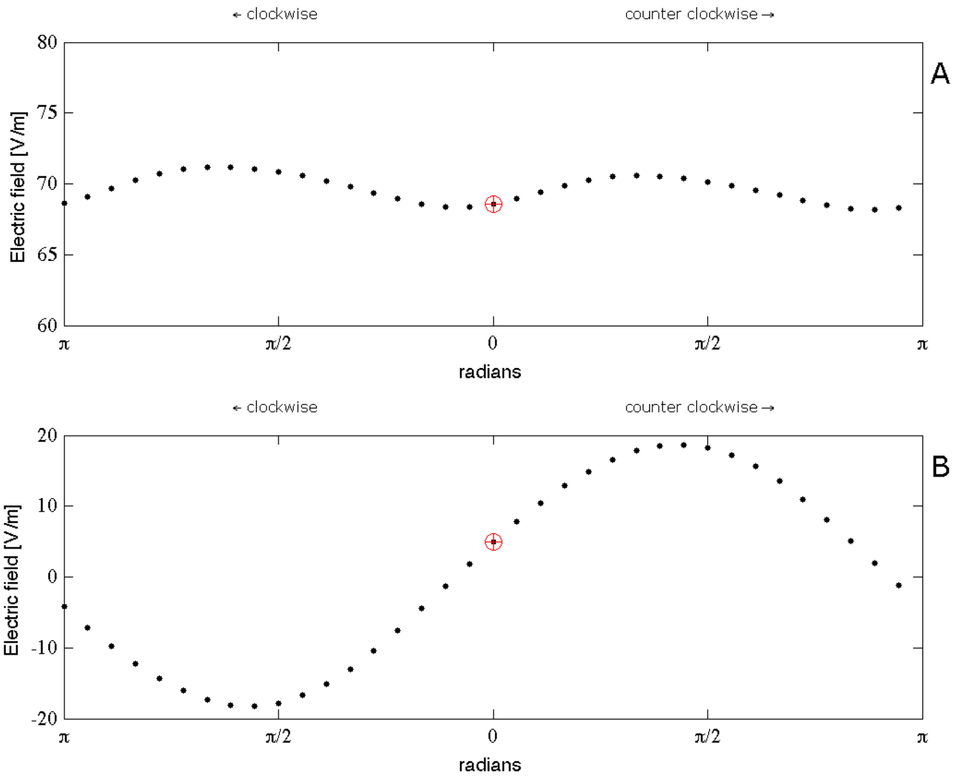

Medial cerebellum left (CM)

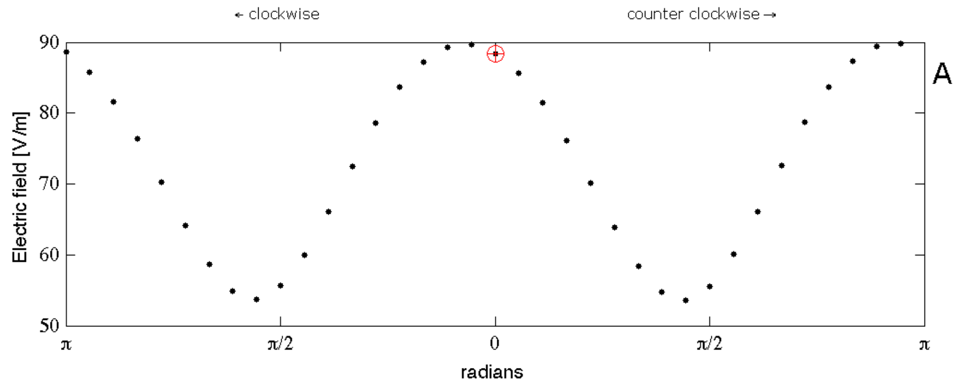

Lateral cerebellum right (CR)

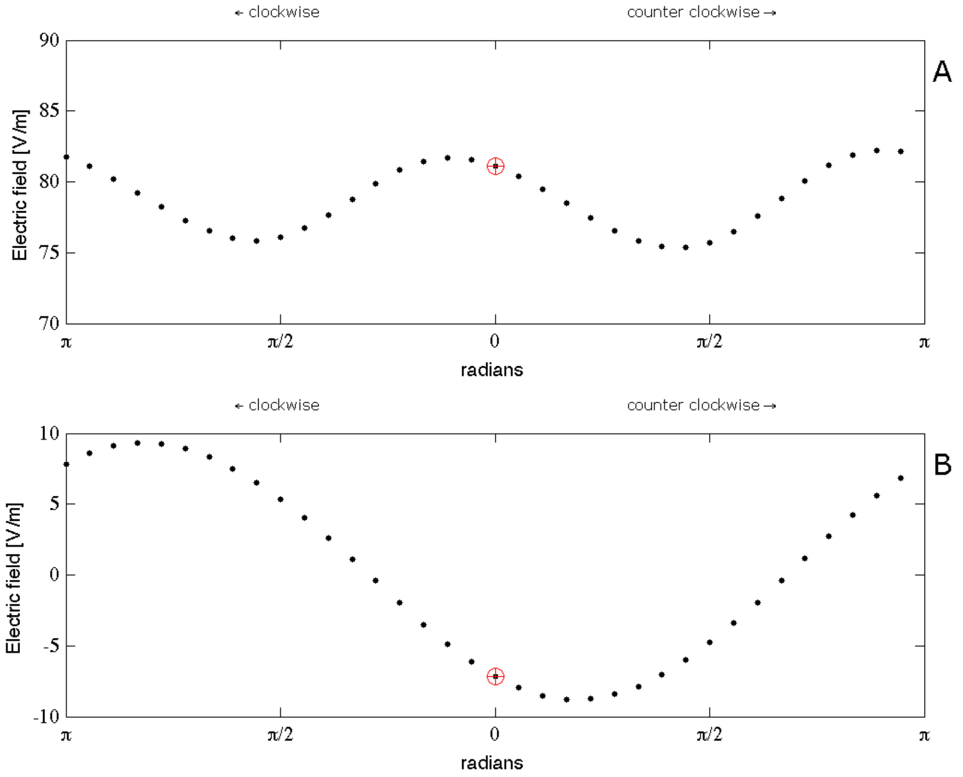

Occipital lobe left hemisphere (OL)

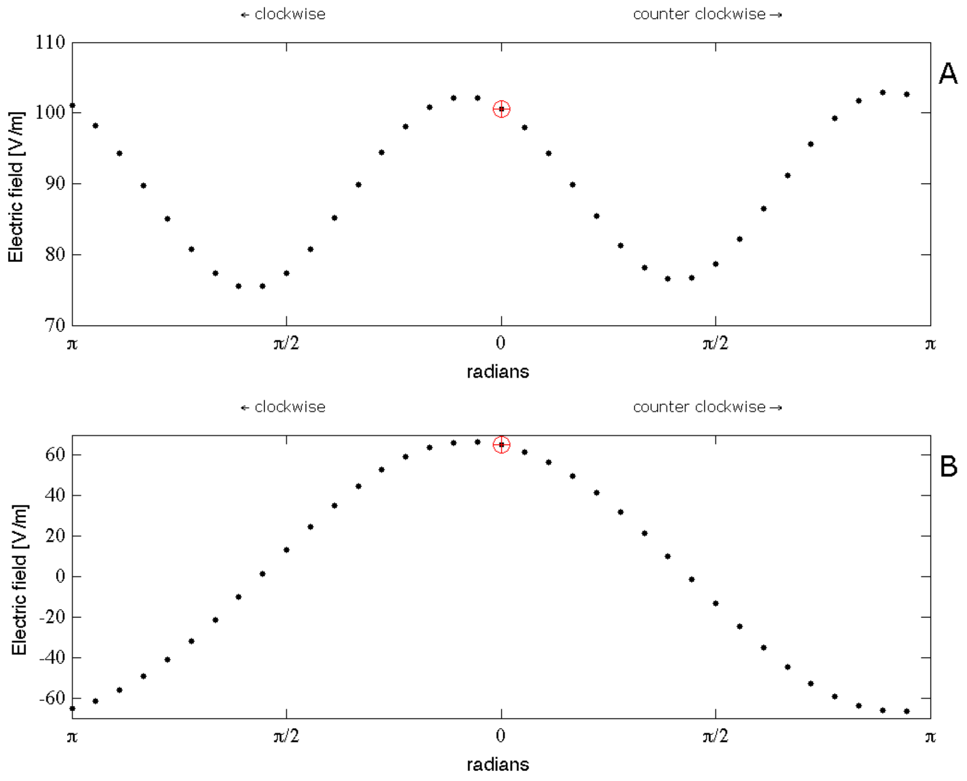

Medial occipital cortex (OM)

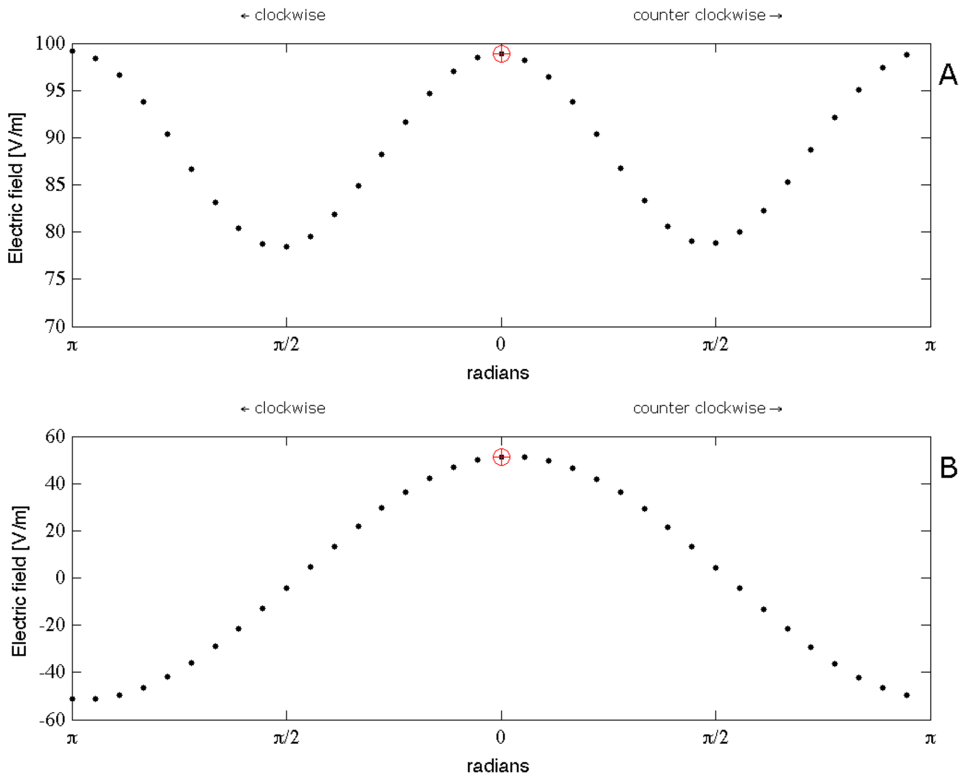

Occipital lobe right hemisphere (OR)

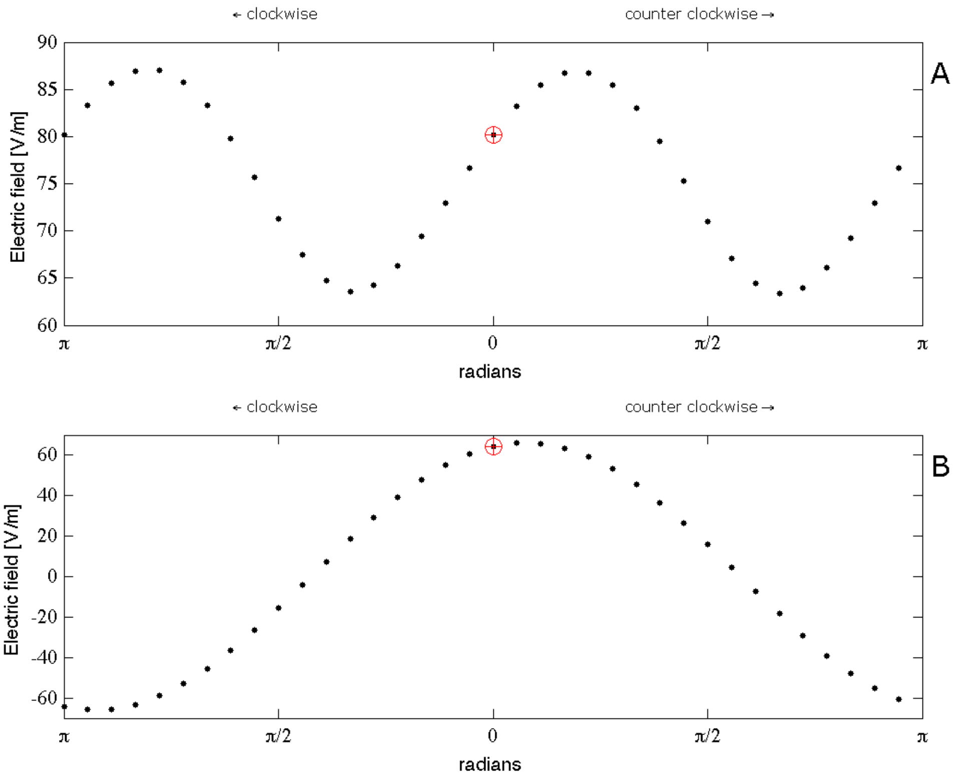

Dorsolateral premotor cortex left hemisphere (PML)

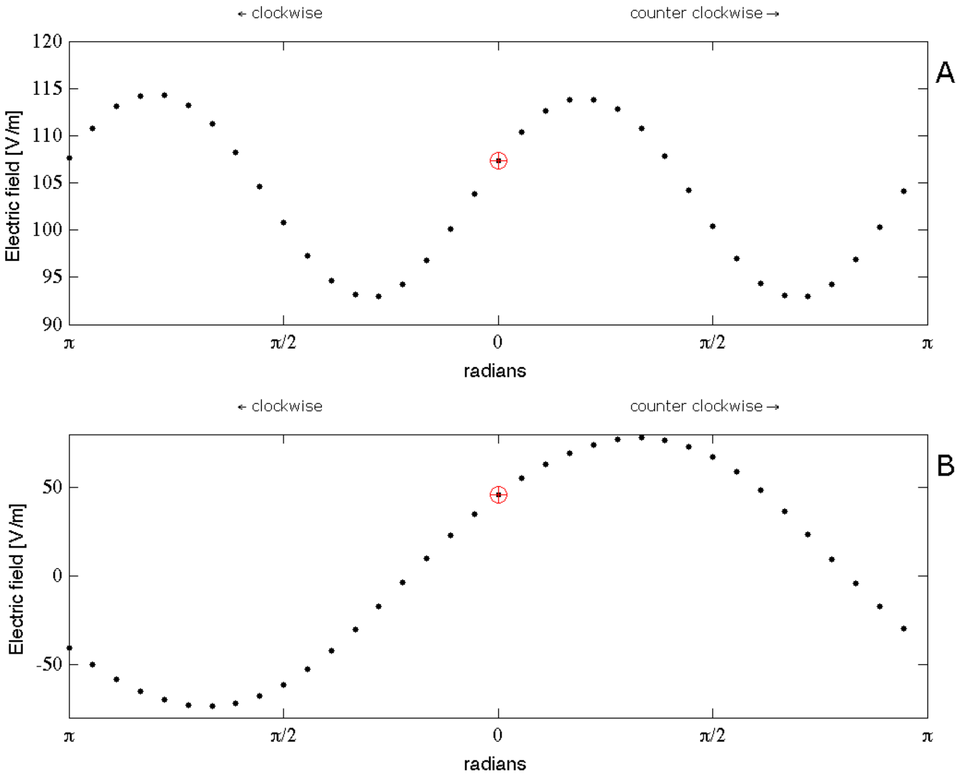

Dorsolateral premotor cortex right hemisphere (PMR)

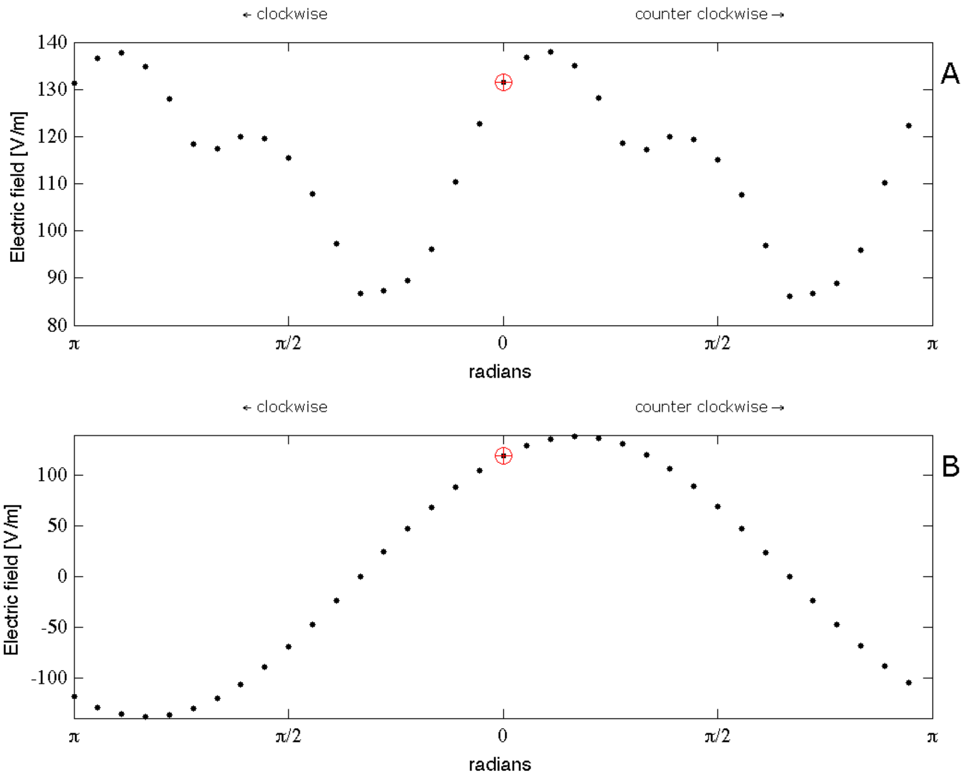

Dorsolateral prefrontal cortex left hemisphere (PFL)

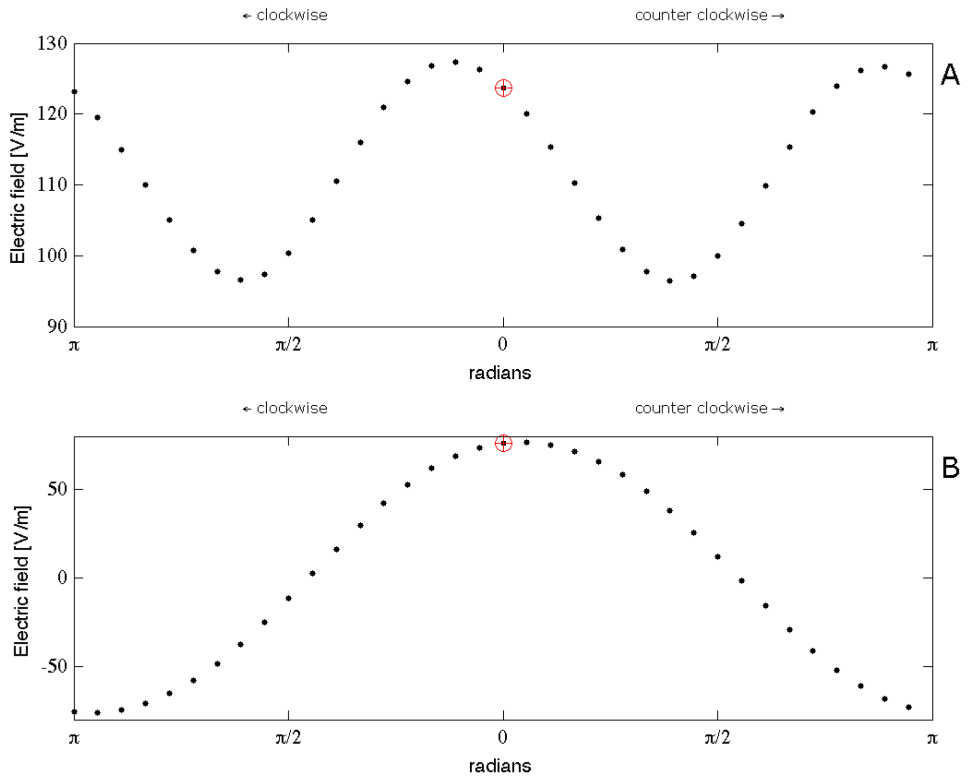

Dorsolateral prefrontal cortex right hemisphere (PFR)

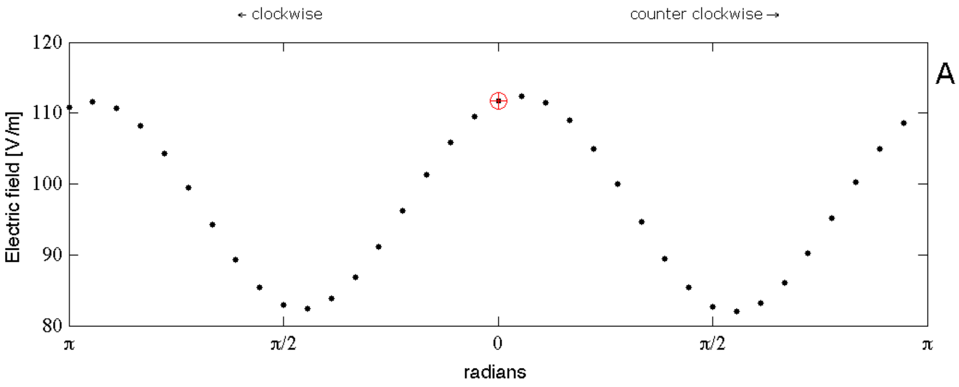

Supplementary motor area 30 mm anterior to Cz (SM1)

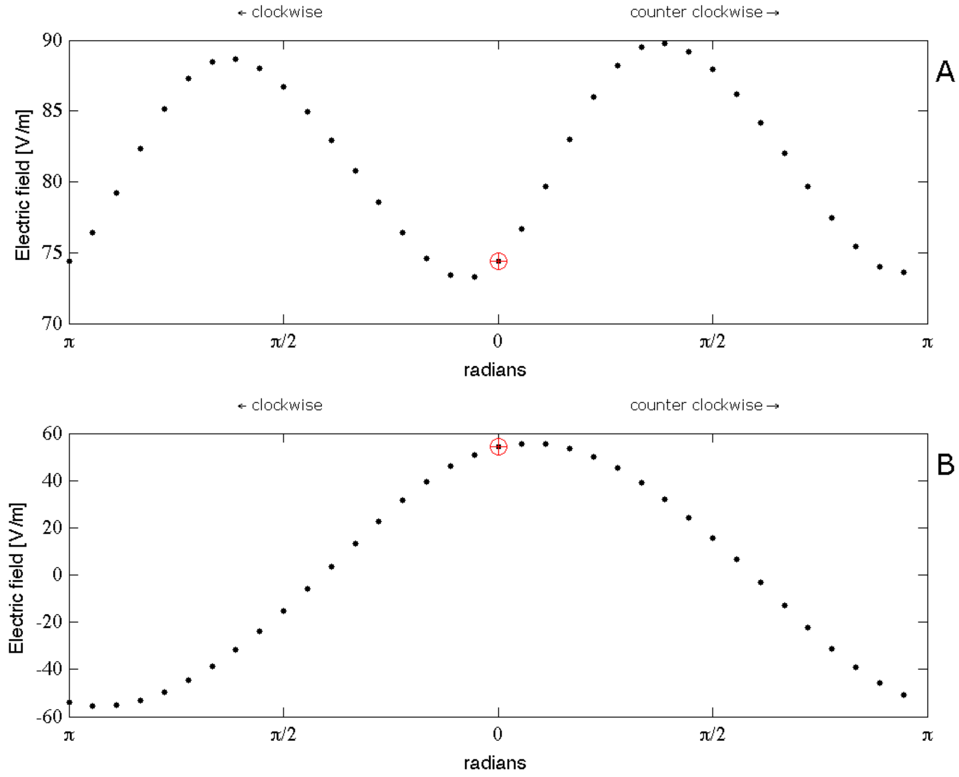

Supplementary motor area 50 mm anterior to Cz (SM2)

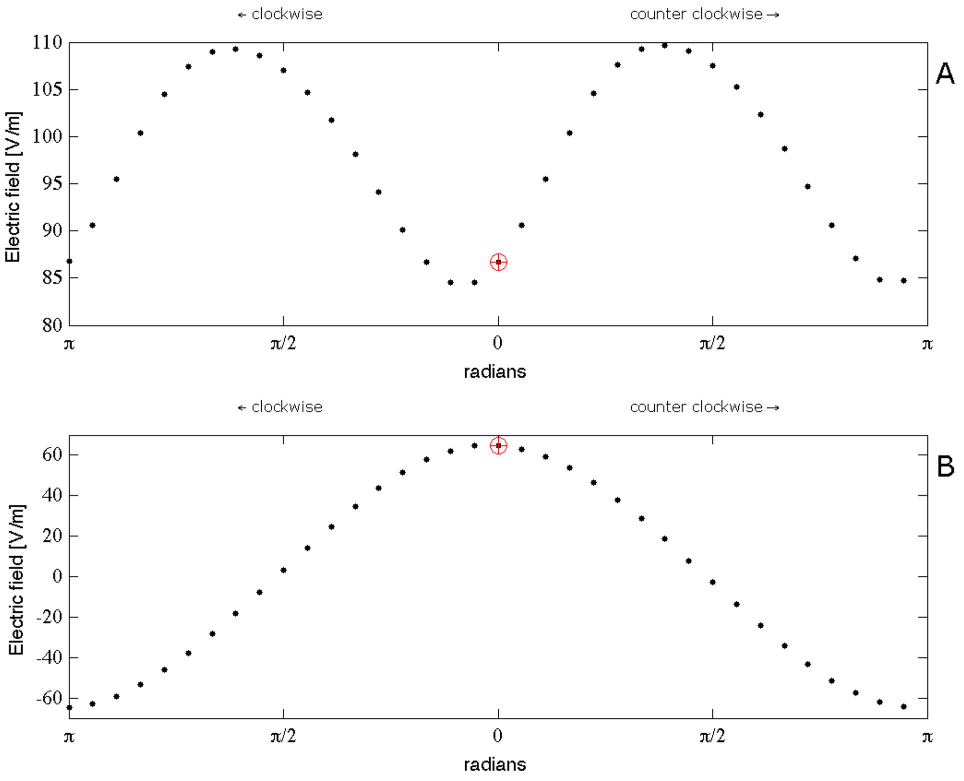

Inferior frontal gyrus (IL)

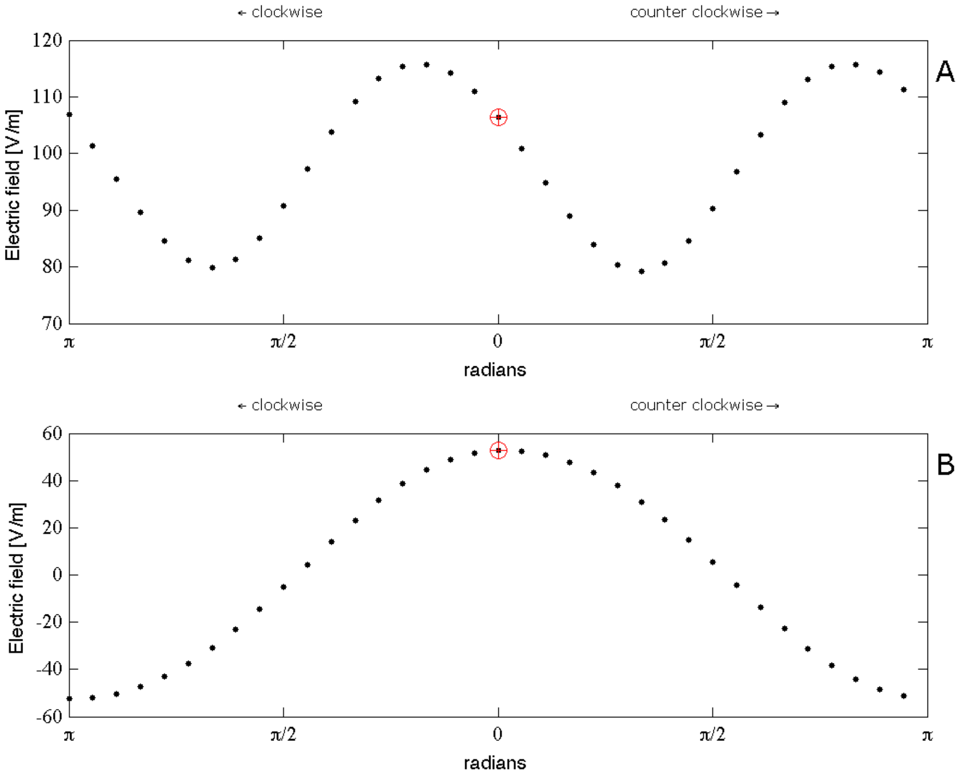

Supplement: Additional file 1: — Mean electric field strength for all target regions. [file 12984_2015_36_MOESM1_ESM.pdf]
